# STAT3-induced upregulation of lncRNA MEG3 regulates the growth of cardiac hypertrophy through miR-361-5p/HDAC9 axis

**DOI:** 10.1038/s41598-018-36369-1

**Published:** 2019-01-24

**Authors:** Jingchang Zhang, Yi Liang, Xuecheng Huang, Xiaoyan Guo, Yang Liu, Jiming Zhong, Jielin Yuan

**Affiliations:** Department of Cardiology, The Third Affiliated Hospital of Guangxi Medical University, Nanning, Guangxi 530031 China

## Abstract

Cardiac hypertrophy is closely correlated with diverse cardiovascular diseases, augmenting the risk of heart failure and sudden death. Long non-coding RNAs (lncRNAs) have been studied in cardiac hypertrophy for their regulatory function. LncRNA MEG3 has been reported in human cancers. Whereas, it is unknown whether MEG3 regulates the growth of cardiac hypertrophy. Therefore, this study aims to investigate the specific role of MEG3 in the progression of cardiac hypertrophy. Here, we found that MEG3 contributed to the pathogenesis of cardiac hypertrophy. MEG3 expression was remarkably strengthened in the mice heart which undergone the transverse aortic constriction (TAC). Moreover, qRT-PCR analysis revealed that MEG3 was upregulated in the cardiomyocytes which were treated with Ang-II. Silenced MEG3 inhibited the increasing size of hypertrophic cardiomyocytes and reversed other hypertrophic responses. Mechanically, MEG3 could affect cardiac hypertrophy by regulating gene expression. Mechanically, we found that MEG3 could be upregulated by the transcription factor STAT3 and could regulate miR-361-5p and HDAC9 by acting as a ceRNA. Finally, rescue assays were made to do further confirmation. All our findings revealed that STAT3-inducetd upregulation of lncRNA MEG3 controls cardiac hypertrophy by regulating miR-362-5p/HDAC9 axis.

## Introduction

Heart failure has become an urgent problem that threatens health of human beings worldwide^1^. Cardiac hypertrophy (CH) is a typical heart disease which caused by increased heart workload, such as aortic stenosis and dilated cardiomyopathy^2,3^. Oversize cardiomyocyte and thick ventricular walls are two main patterns of cardiac hypertrophy^2,3^. Maladaptive hypertrophy was featured as increased size of cardiomyocyte and activation of fibroblast. This disease is closely associated with weakened systolic function and heart rigidity^4^. Left ventricular hypertrophy has strong correlation with an growing risk of adverse cardiovascular events^5^. Accumulating reports have shown the crucial functions of lncRNAs in the progression of cardiac hypertrophy. For instances, lncRNA MIAT motivated cardiac hypertrophy via modulation of miR-93/TLR4^6^; LncRNA TINCR could attenuate cardiac hypertrophy via epigenetically silencing CaMKII^7^; LncRNA-ROR reconciled the reprogramming in Cardiac Hypertrophy^8^. All these reports provided evidences for the special role of lncRNAs in CH.

Long non coding RNA maternally expressed gene 3 (MEG3) is a typical lncRNA which could modulate biological process in human diseases, such as various human cancers^9–11^ and other common diseases^12,13^. This study aims to explore the biological function of MEG3 in CH. MEG3 was found to be upregulated in both TAC group and Ang-II group. Therefore, we silenced MEG3 in to do functional assays. The oversize cardiomyocytes caused by Ang-II partially reversed after MEG3 was knocked down. Afterwards, the molecular mechanism by which MEG3 exert its functions in cardiac hypertrophy was investigated. STAT3 was demonstrated to be the upstream transcription factor of MEG3. Furthermore, MEG3 can act as a ceRNA to regulate miR-361-5p and HDAC9 in cardiac hypertrophy. In conclusion, this study focused on the mechanism and function of STAT3-MEG3-miR-361-5p-HDAC9 axis in cardiac hypertrophy.

## Materials and Methods

### Animals and histological analysis

Vital River Laboratory Animal Company (Beijing, China) provided C57BL6 mice (SPF, male, 18–25 g, 8 weeks) for this experiment. All experiments were conducted in accordance with the principles approved by the institutional Animal Care and Use Committee of The Third Affiliated Hospital of Guangxi Medical University. The experimental procedures were conducted abiding by the Care and Use of Laboratory Animals published by The Third Affiliated Hospital of Guangxi Medical University. A total of 10 CH mouse models used in this study were induced by transverse aortic constriction (TAC). This step conformed to a previous study^8^. In brief, intraperitoneal ketamine (85 mg/kg) and xylazine (10 mg/kg) were used to anesthetize the mice. Then, the transverse thoracic aorta of the mice was dissected and the surgery silk thread and a 26-gage needle were used to suture the wound. The sham group was generated in the age-matched mice whose aorta was not sutured. All mice recovered on a ventilator at 37 °C after the operation. One week later, the hearts of mice were collected for further analysis.

### Cardiomyocyte culture and treatment

Cardiomyocytes were isolated from newborn mice hearts as previously described^14,15^. After the quick washing, the isolated hearts were sliced into some small pieces. Then the tissues were transferred into HEPES-buffered saline solution complex (0.1% trypsin +0.14 mg/ml collagenase) (Roche, USA) at 37 °C. Dulbecco’s modified Eagle’s medium/F12 (Invitrogen, USA) was utilized for re-suspending the dissociated cells. After centrifugation, cardiomyocytes were pre-plated at 37 °C for 1 h for isolation. Next, cardiomyocytes were collected for the next experiments. The expression level of MEG3 was treated with different dose of angiotensin II (Ang-II, Sigma, USA). The highest expression of MEG3 was observed in 150 nM. Therefore, subsequent experiments were carried out in cardiomyocytes which were treated with 150 nM of Ang-II.

### Immunofluorescence staining and cell surface area assay

At first, use the cold PBS to wash cultured cardiomyocytes for three times. Then, 4% paraformaldehyde was utilized to fix cells for 15 min. Cardiomyocytes were then washed with cold PBS again so that the cardiomyocytes could be blocked with 10% normal goat serum and 1% BSA (Sigma, USA). Next, cardiomyocytes were incubated with anti-α-actinin (1:500, Sigma, USA) in a dark room at 4 °C overnight and were further incubated with secondary antibody (1:1000; Molecular Probes, USA) at a normal temperature for 1 hour. Next, the slides were covered with coverslips. The nucleus was stained with mounting medium (Abcam, USA) which containing Hoechst33342 (Beyotime, China). At length, a fluorescence microscope (Olympus, Japan) helped us to observe and photograph cells. Cell surface area was quantified by detecting 50 random cells from three independent experiments. Thereafter, the average value was used for further analysis. Cell surface areas were assessed by ImageJ software by observing the 100–200 cardiomyocytes in 30–40 fields.

### Quantitative real-time PCR

Total RNA isolation from heart tissues and cultured cardiomyocytes was conducted using Trizol reagent (Invitrogen, USA). Reverse transcription was finished by using a Prime-Script RT reagent kit (Takara, Dalian, China). For amplification and detection of required genes, a TaqMan Reverse Transcription kit (Takara, Dalian, China) was used for synthesizing cDNA. The expression levels for lncRNA, miRNA and mRNA were relative to the levels of GAPDH or U6. qRT-PCR was carried out on an ABI 7500 fast Real-Time PCR system by the use of PowerSYBR Green PCR master mix (Applied Biosystems, USA). The relative expression of each gene was calculated using the 2^−ΔΔCt^ method.

### Northern blot assay

Total RNAs were extracted from cardiomyocytes using the TRIzol reagent (Invitrogen, CA, USA) and quantified by spectrophotometer method. The total RNA was kept at −80 °C. After separating on 7% PAGE gels, RNAs were transferred onto Magna NT nylon membranes, followed by baking at 50 °C for 30 min and 2 cycles of UV light crosslink (Spectronics Corp, New York, USA). Northern hybridization was conducted in line with supplier’s direction. Membranes were rinsed, blotted dry and treated with a Phosphor Imager screen. The hybridization bands were quantitated using Image Quant software.

### Plasmid construction and transfection

To overexpress MEG3 and HDAC9, MEG3 cDNA and HDAC9 cDNA were cloned into pCDNA3.1 vector (Invitrogen, USA). To increase or decrease the expression level of miR-361-5p, miR-361-5p mimics and miR-361-5p inhibitors were separately transfected into cells. Both of them and negative control miRNA were bought from RiboBio Co. (Shanghai, China). For knockdown of MEG3 or STAT3, shRNAs targeting to MEG3 (sh-MEG3) or STAT3 (sh-STAT3) and NC-shRNAs were synthesized and bought from RiboBio Co. (Shanghai, China). Based on the user guide, all transfections were finished by using Lipofectamine2000 (Invitrogen, USA). Cells were harvested after transfection for two days.

### Cell cytoplasm/nucleus fraction isolation

NE-PER Nuclear and Cytoplasmic Extraction Reagents (Thermo Scientific, Waltham, MA, USA) were used for extraction of cytoplasmic and nuclear fractions from cardiomyocytes. qRT–PCR analysis was applied to analyze RNAs extracted from each of the fractions, and examine the levels of nucleus and cytoplasm of lncRNA MEG3.

### Chromatin immunoprecipitation (ChIP) assay

The Magna ChIP Kit (Millipore, Bedford, MA, USA) was utilized for ChIP assays. To generate the DNA-protein cross-links, cardiomyocyte was treated with formaldehyde. The chromatin fragments were generated by sonicating the cell lysates. Next, the lysates were immunoprecipitated with antibody (Millipore). IgG was used as a control. qPCR was used to recover the precipitated chromatin DNA.

### RNA-binding protein immunoprecipitation

In accordance with the protocol of manufacturer, the Magna RIP RNA-binding protein immunoprecipitation kit (Millipore, Billerica, MA, USA) and the Ago2 antibody (Abcam, Cambridge, MA, USA) were utilized for performing RIP experiments. Co-precipitated RNAs were analyzed by means of qRT-PCR analysis.

### RNA pull-down assay

Cardiomyocytes were transfected with biotinylated miRNA. Two days later, cells were collected for next steps. M-280 streptavidin magnetic beads (Invitrogen) was utilized for incubation of the cell lysates. We purified the bound RNAs with TRIzol reagent (Invitrogen) for qRT–PCR analysis.

### Dual luciferase reporter assay

Cells were seeded in 24-well plates (3 × 10^4^ cells/well). The next day, the cells were co-transfected with pmirGLO-MEG3-WT or pmirGLO-MEG3-MUT reporter plasmids and miR-361-5p mimics. The sequence of MEG3 containing the predicted binding site with miR-361-5p; then we cloned the putative and mutated sequences of the binding site into a pmirGLO Dual-luciferase miRNA target expression vector to shape the reporter vector pmirGLO-MEG3 -wild type (MEG3-WT) and pmirGLO-MEG3 -mutated type (MEG3-MUT). Lipofectamine 2000 was taken advantage of co-transfecting miR-142-3p mimics or miR-NC into the pmirGLO-MEG3-WT or pmirGLO-MEG3-MUT in cells. Two days after transfection, Dual-Luciferase Reporter Assay System (Promega, Madison, WI, USA) enabled us to measure the relative luciferase activity.

### Western blot analysis

Proteins were lysed from heart tissues and cells with RIPA buffer (Beyotime, China) on ice and they were centrifuged at 4 °C for 20 min. Protein concentration was determined with a BCA protein assay kit (Beyotime, China). Subsequently, SDS-PAGE was utilized to separate proteins (30 μg each lane). Then proteins were transferred onto PVDF membranes (Millipore, USA). Afterwards, 5% defatted milk was used to block the membranes in TBST buffer. Next, the membranes were exposed to primary antibodies at 4 °C overnight. After washing, the membranes were incubated with secondary antibodies for 45 min at normal temperature. GAPDH was used as an internal control. Finally, an enhanced chemiluminescence detection system (Pierce, Rockford, USA) was used to detect proteins.

### Statistical analysis

Data were displayed as the mean ± SD from at least three independent experiments. All experiments were independently conducted at least three times. Statistical significance between two groups was determined with the Student’s t test. Experimental data among multiple groups were compared and analyzed with One-way ANOVA (SPSS 20.0). Expression correlation was analyzed by Spearman’s correlation analysis. Differences were considered to be statistically significant only when P value less than 0.05.

## Results

### MEG3 plays a positive role in CH *in vivo*

**Figure 1:**
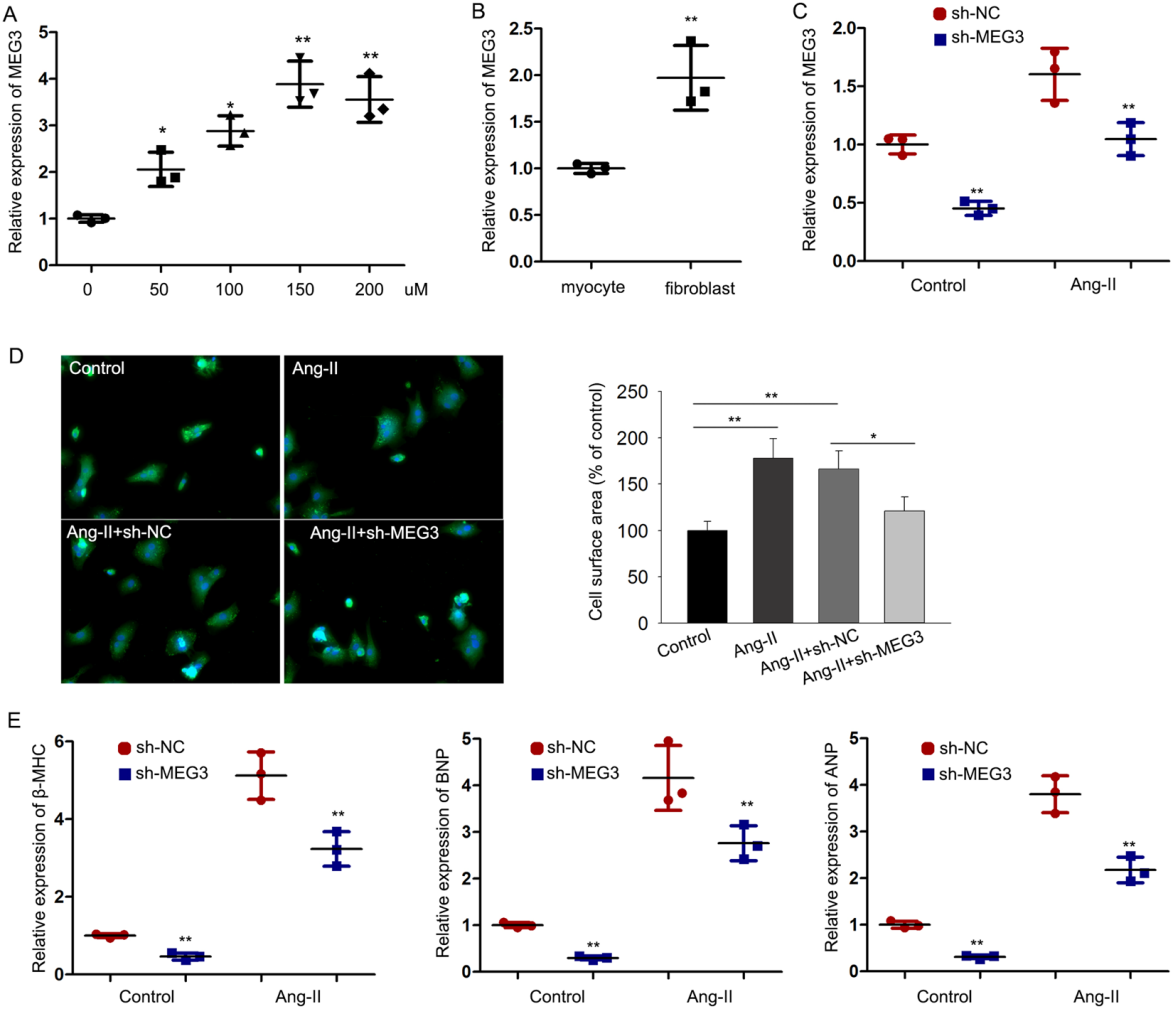
MEG3 plays a positive role in CH *in vivo*. (**A**) qRT-PCR was performed to examine relative expression of MEG3 under the different dose of Ang-II (0, 50, 100, 150, 200 nM) for about 48 hours (n = 3 per group; ^*^p < 0.05; ^**^p < 0.01). The results were normalized to GAPDH. (**B**) The level of MEG3 expression was detected in different cardiac cells (myocyte and fibroblast) (n = 3 per group; ^**^p < 0.01). (**C**) shRNA was used to silence MEG3 expression in both control group and Ang-II group (n = 3 per group; ^**^p < 0.01). The transfection efficiency was measured after 48 hours. (**D**) After transfection for 48 hours, immunofluorescence staining with anti-α-actin was performed to detect the influence of silenced MEG3 on the size of cardiomyocytes induced by Ang-II group (n = 3 per group; ^**^p < 0.01, N.S: no significance). The cell size was measured in 10 fields/well in both groups. Scale bar = 100 μm. (**E**) The expression of three elements (β-MHC, ANF and BNP) was examined after MEG3 was down-regulated in both control group and Ang-II group (n = 3 per group; ^**^p < 0.01).

In has been reported that the abnormal expression of MEG3 is closely related to the occurrence and progression of human diseases^11,16^. This study aims to explore the special function of MEG3 in cardiomyocyte hypertrophy. According to previous report, we knew that angiotensin II (Ang-II) can induce CH^17^. Here, we treated the cardiomyocytes with Ang-II. qRT-PCR was used to explore whether the expression of MEG3 in cardiomyocytes can be affected by the dose change of Ang-II (0, 50, 100, 150, 200 nM). As shown in Fig. 1A, the highest level of MEG3 was tested when cardiomyocytes were treated with 150 nM Ang-II. Therefore, we chose this dose of Ang-II to do next experiments. To obtain adequate evidence, we detected MEG3 expression in different cardiac cells (without Ang-II). As a result, the expression of MEG3 was more abundant in fibroblast than that in myocyte (Fig. 1B). Next, MEG3 was silenced in cardiomyocytes which were treated with or without Anh-II by specific shRNAs (sh-MEG3) and control shRNA (sh-NC) (Fig. 1C). The transfection efficiency was obtained after 48 hours. Immunofluorescence staining with anti-α-actin was used to detect the influence of silenced MEG3 on the cell surface area of cardiomyocytes. It was uncovered that Ang-II-induced bigger cell surface area was reversed by sh-MEG3 (Fig. 1D). Atrial natriuretic factor (ANF), brain natriuretic peptide (BNP), and β-myosin heavy chain (β-MHC) are three hypertrophic markers. Upregulation of these three markers in cardiomyocytes are taken as common cardiac hypertrophy responses. The expression levels of β-MHC, ANF and BNP were decreased by sh-MEG3 (Fig. 1E). All the above findings suggested that MEG3 positively regulates cardiac hypertrophy.

### MEG3 is upregulated by the transcription factor STAT3

**Figure 2:**
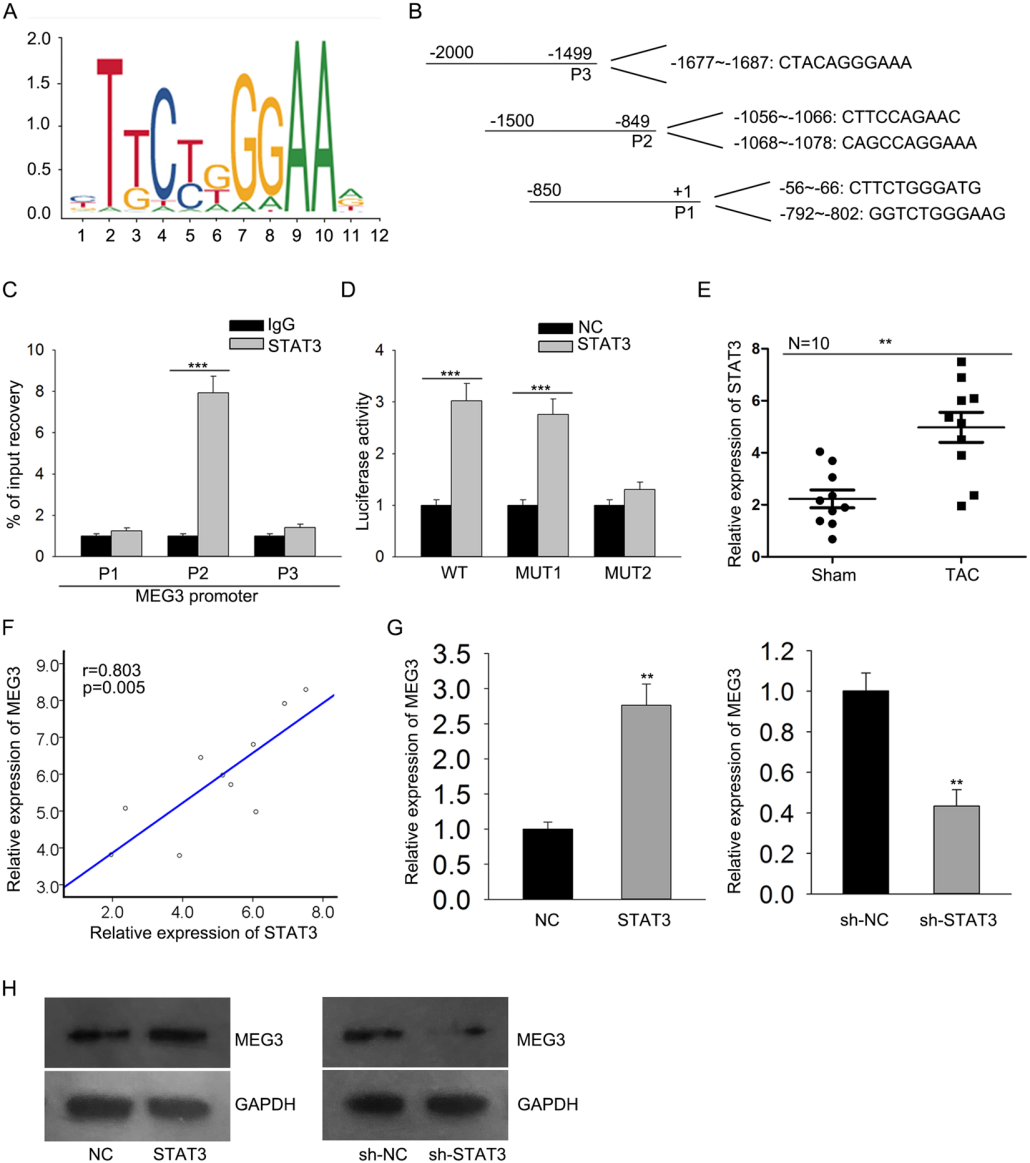
MEG3 is upregulated by the transcription factor STAT3. (**A**) The binding motif of STAT3 was obtained from JASPAR. (**B**) Top five binding sequences of STAT3 to three parts of MEG3 promoter were shown. (**C**) ChIP analysis revealed the affinity of STAT3 to the part 2 (P2) of MEG3 promoter (^***^p < 0.001). (**D**) Luciferase activity analysis was used to further confirm the binding of STAT3 to MEG3 promoter (^***^p < 0.001). (**E**) The level of STAT3 was then examined in ten pairs of mouse hearts treated with Sham or TAC (^**^p < 0.01). (**F**) The expression correlation between STAT3 and MEG3 in TAC group was analyzed by Spearman’s correlation analysis (^**^p = 0.005). (**G**,**H**) The expression of MEG3 in cardiomyocytes was detected after STAT3 was upregulated or downregulated (n = 3 per group; ^**^p < 0.01).

Based on the data of Fig. 1, high expression MEG3 might be a promoter in cardiac hypertrophy. Here, we investigated the molecular mechanism underlying MEG3 upregulation. The search result of UCSC (http://genome.ucsc.edu) suggested that STAT3 was found to be the upstream transcription factor of MEG3. The binding motif of STAT3 was obtained from JASPAR (http://jaspar.genereg.net/) and illustrated in Fig. 2A. Top five bindings sequences of STAT3 to three parts of MEG3 promoter were shown in Fig. 2B. Subsequently, ChIP analysis revealed the affinity of STAT3 to the part 2 (P2) of MEG3 promoter (Fig. 2C). Luciferase activity analysis was used to make further confirmation. The result indicated that the luciferase activity of vector containing mutated biding site 2 (−1068~−1078) was not increased by STAT3 (Fig. 2D), indicating the binding relation between STAT3 and MEG3 promoter in site 2. The level of STAT3 was then examined in ten pairs of smouse hearts treated with Sham or TAC. Not surprisingly, STAT3 was expressed higher in TAC group (Fig. 2E). The positive expression correlation between STAT3 and MEG3 in TAC group was analyzed by Spearman’s correlation analysis (Fig. 2F). Furthermore, qRT-PCR and northern blot assays revealed that the expression of MEG3 in cardiomyocytes was positively regulated by STAT3 (Fig. 2G,H), indicating the positive regulatory effect of STAT3 on the expression of MEG3.

### MEG3 serves as a miR-361-5p sponge

**Figure 3:**
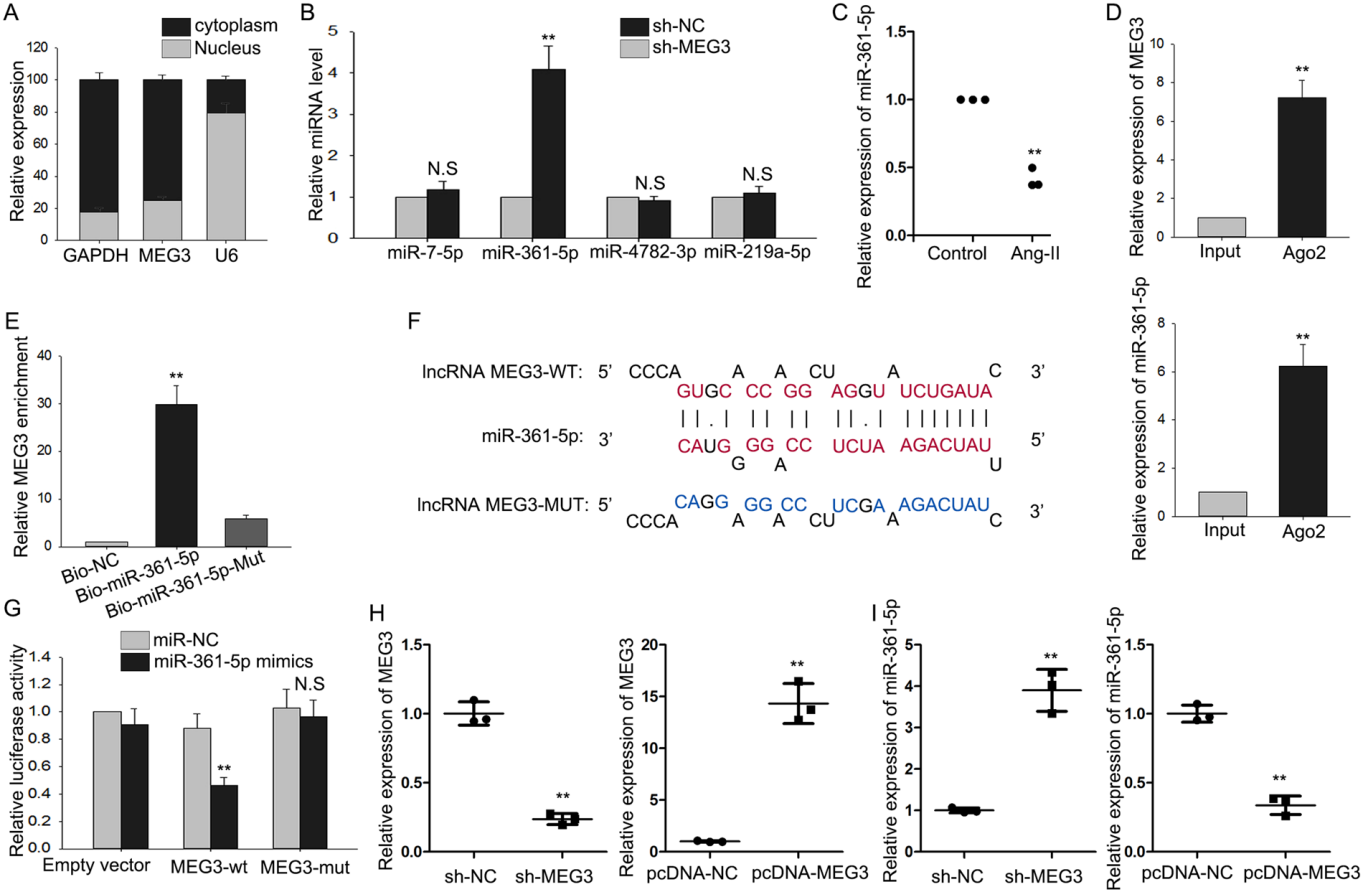
MEG3 serves as a miR-361-5p sponge. (**A**) Subcellular fractionation assay was conducted in CH cell to detect the localization of MEG3. (**B**) The expression levels of four miRNAs which could bind with MEG3 were tested in MEG3 downregulated CH cells (^**^p < 0.001; N.S: no significance). (**C**) The expression of miR-361-5p was detected in cardiomyocytes treated with or without Ang-II by qRT-PCR (n = 3 per group; ^**^p < 0.01). (**D**) RIP assay was carried out to demonstrate the combination between MEG3 and miR-361-5p (^**^p < 0.01). (**E**) Pull down assay was applied to prove the binding relation between MEG3 and miR-361-5p (^**^p < 0.01). (**F**) The binding sequence between miR-361-5p and MEG3-WT/MUT was predicted. (**G**) Luciferase reporter assay was conducted to detect the luciferase activity of MEG3-WT/MUT after transfection with miR-361-5p mimics (^**^p < 0.01; N.S: no significance). (**H**) MEG3 was silenced or overexpressed with shRNA or pcDNA3.1 vector (n = 3 per group; ^**^p < 0.01). qRT-PCR was used to detect the transfection efficiency after 48 hours. (**I**) The expression levels of miR-361-5p were tested in MEG3-downregulated or MEG3-overexpressed cells with qRT-PCR (n = 3 per group; ^**^p < 0.01).

It has been reported that lncRNAs can act as a ceRNA in human diseases by competitively sponging miRNAs^18,19^. In this study, we hypothesized that MEG3 exert function of ceRNA in cardiac hypertrophy. At first, we applied subcellular fractionation assay to detect the specific localization of MEG3 in cardiomyocytes. It was found to be mainly located in the cytoplasm (Fig. 3A). Next, we found out four miRNAs which can bind with MEG3 from starbase (http://www.lncrnablog.com/starbase-v2–0-for-decoding-rna-interaction-networks/). Next, we examined the expression level of all these four miRNAs in response to the silencing of MEG3. As a result, only miR-361-5p was obviously upregulated (Fig. 3B). Therefore, miR-361-5p was chosen to do next experiment. Next, miR-361-5p was found to be upregulated in cardiomyocytes which were treated with Ang-II (Fig. 3C). RIP assay revealed that both MEG3 and miR-361-5p tended to enrich in Ago2-immunoprecipitation (Fig. 3D). What’s more, RNA pull down assay was conducted and indicated that MEG3 could only be pulled down by the wild type bio-labeled miR-361-5p, but not the mutated oligos (Fig. 3E). The binding sites between MEG3 and miR-361-3p was shown in Fig. 3F. Dual luciferase reporter assay was applied to further demonstrate the combination between them. After miR-361-5p and miR-NC were separately transfected into cardiomyocytes, we found that only the luciferase activity of wild type MEG3 (MEG3-WT) was obviously changed by miR-361-5p mimics (Fig. 3G). Next, MEG3 was overexpressed by pcDNA-MEG3 but was silenced by sh-MEG3 (Fig. 3H). The transfection efficiency was harvested after 48 hours. The expression levels of miR-361-5p were negatively regulated by MEG3 (Fig. 3I). Accordingly, we confirmed that MEG3 acts as a ceRNA by competitively binding with miR-361-5p in cardiac hypertrophy.

### MEG3 upregulates HDAC9 through competitively binding with miR-361-5p

**Figure 4:**
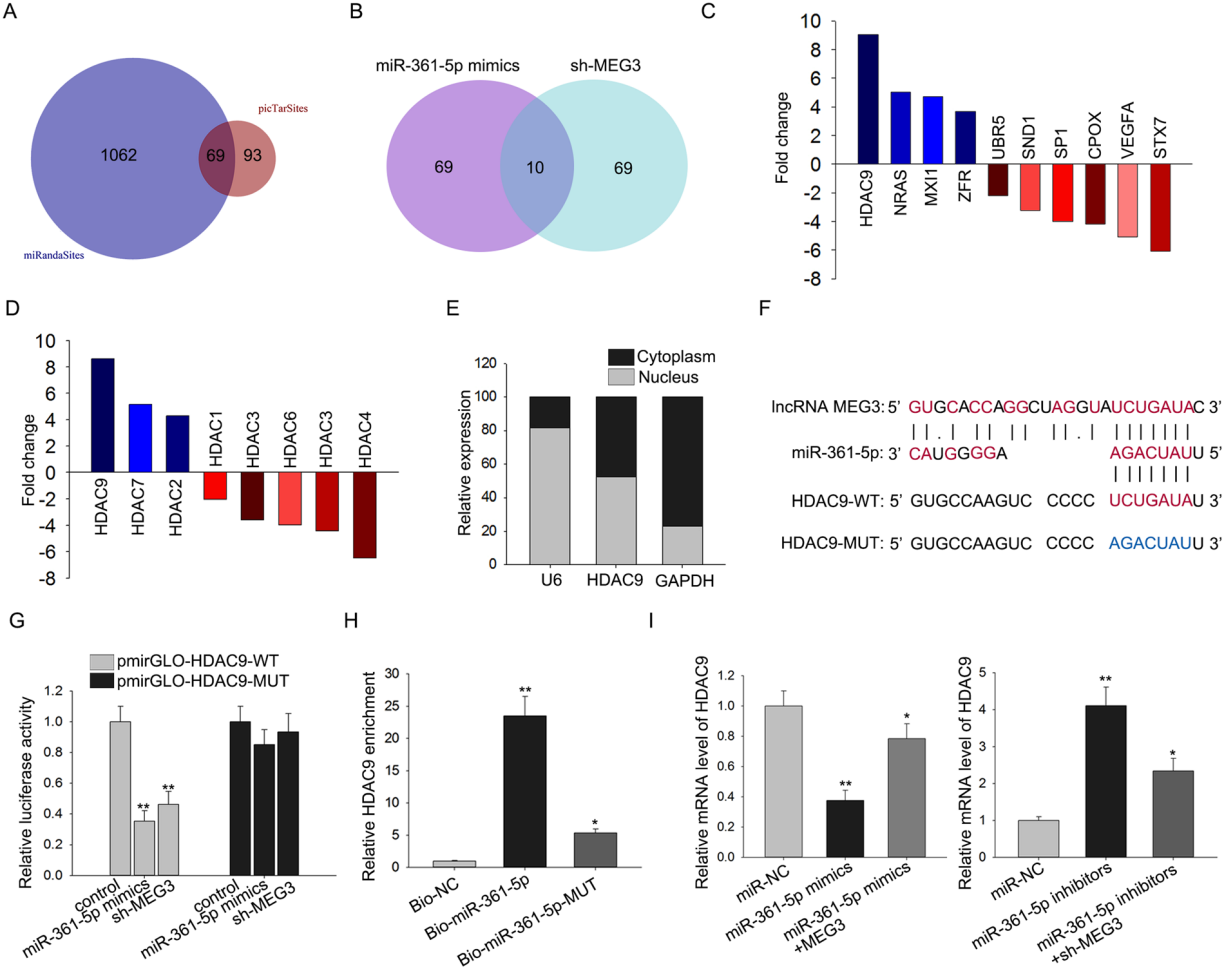
MEG3 upregulates HDAC9 through competitively binding with miR-361-5p. (**A**) 69 potential target mRNAs of miR-361-5p was predicted from two public bioinformatics websites (picTarSites and miRandaSites). (**B**) The expression level of all these 69 mRNAs was examined in response to miR-361-5p mimics and sh-MEG3. (**C**) The fold change of all 69 candidate mRNAs was examined. (**D**) The fold change of HDACs family (HDAC1, HDAC2, HDAC3, HDAC5, HDAC6, HDAC7 and HDAC9) was detected. (**E**) The localization of HDAC9 was examined in cardiomyocytes by subcellular fractionation. (**F**) The binding sites between MEG3 and miR-361-5p as well as between miR-361-5p and HDAC9 was predicted by using bioinformatics analysis. (**G**) Luciferase reporter assay was applied to determine the effects of miR-361-5p mimics or sh-MEG3 on the luciferase activities of HDAC9-WT/MUT (^**^p < 0.01). (**H**) Pull down assay was conducted to prove the combination between miR-361-5p and HDAC9 (^*^p < 0.05, ^**^p < 0.01). (**I**) The effects of MEG3 and miR-361-5p on the expression levels of HDAC9 were detected (n = 3 per group; ^*^p < 0.05, ^**^p < 0.01).

Bioinformatics analysis was applied to find the mRNA which constitutes a ceRNA model with miR-361-5p and HDAC9 in CH. We found 69 potential target mRNAs of miR-361-5p by using two public bioinformatics tools (picTarSites and miRandaSites) (Fig. 4A). Subsequently, the expression of these 69 mRNAs was examined in response to miR-361-5p mimics and sh-MEG3. As a result, only ten of them was obviously downregulated (Fig. 4B). Therefore, these ten mRNAs were chosen for further study. Next, the fold change of all these ten mRNAs in the mouse hearts in TAC and Sham group (Fig. 4C). HDAC9 showed the most obvious fold change. In addition, we detected the fold change of HDACs family (HDAC1, HDAC2, HDAC3, HDAC5, HDAC6, HDAC7 and HDAC9). As a result, HDAC9 exhibited the most obvious fold change (Fig. 4D). Moreover, HDAC9 has been proved to be targeted by miRNA in human disease^20^. Here, we supposed that HDAC9 can participate in a ceRNA model. To further demonstrate our hypothesis, the localization of HDAC9 was examined in cardiomyocytes. It was uncovered to be located in both nucleus and cytoplasm (Fig. 4E). Next, the binding sites between MEG3 and miR-361-5p as well as between miR-361-5p and HDAC9 was predicted by using bioinformatics analysis (Fig. 4F). The result of luciferase reporter assay manifested that the luciferase activities of HDAC9-WT was largely decreased by miR-361-5p mimics or sh-MEG3 (Fig. 4G). Pull down assay was conducted and suggested that HDAC9 could only be pulled down by the wild type bio-labeled miR-361-5p, but not the mutated oligos (Fig. 4H). The decreased expression level of HDAC9 caused miR-361-5p mimics was rescued by pcDNA-MEG3 (Fig. 4I). The expression level of HDAC9 increased by miR-361-5p inhibitors was partially reversed by sh-MEG3 (Fig. 4I). All these experimental results indicated that MEG3 can positively regulate HDAC9 via sponging miR-361-5p.

### The relationships among MEG3, miR-361-5p and HDAC9

**Figure 5:**
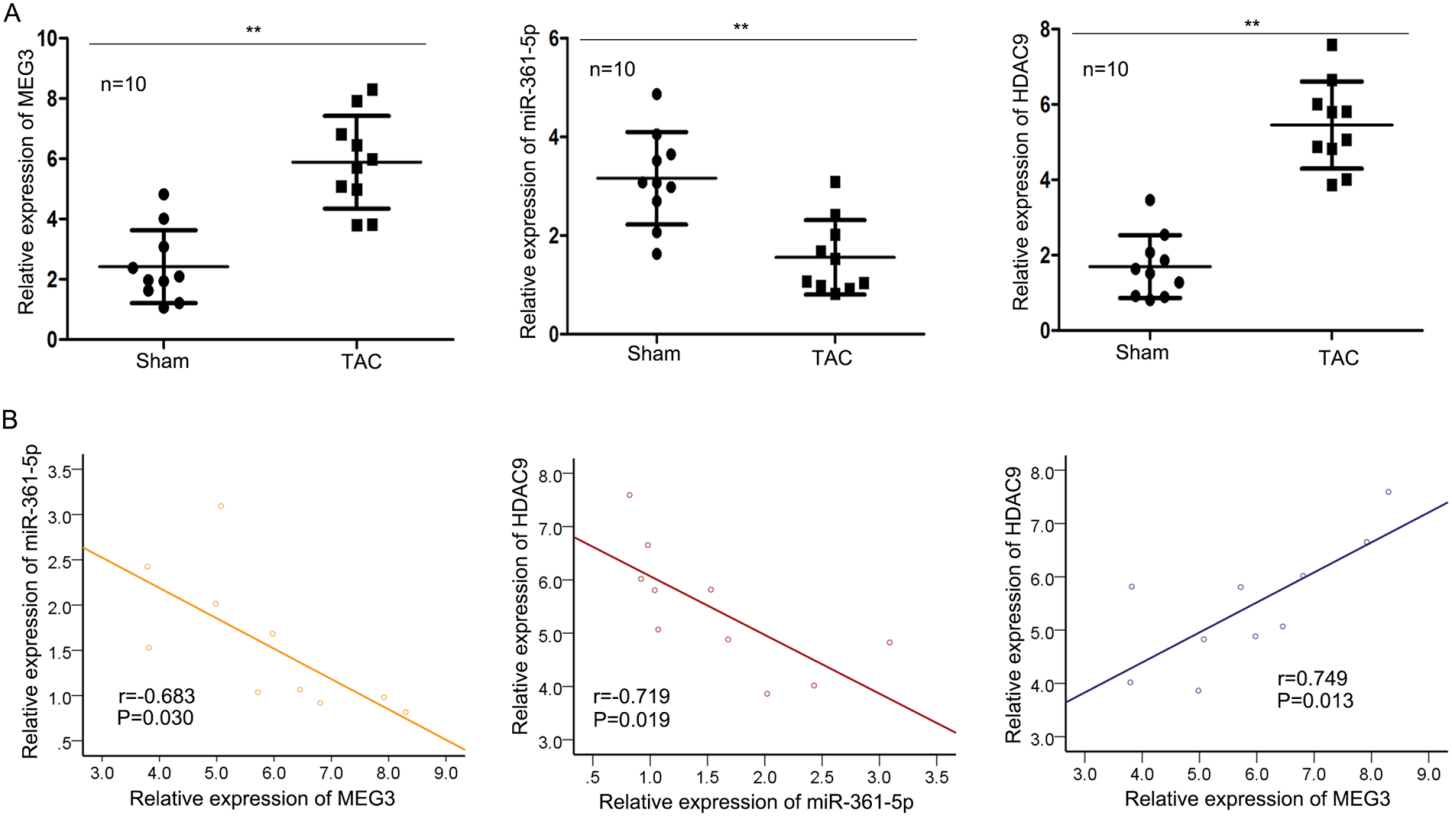
The relationships among MEG3, miR-361-5p and HDAC9. For this study, mouse models treated with Sham and TAC were constructed. They were harvested after one weeks. (**A**) The expression profiles of MEG3, miR-361-5p and HDAC9 were examined in paired mouse hearts treated with Sham and TAC by qRT-PCR (n = 10 per group; ^**^p < 0.01). (**B**) Spearman’s correlation method was utilized to analyze the correlations among MEG3, miR-361-5p and HDAC9 (^*^p < 0.05).

To understand whether MEG3 is differently expressed in CH samples, we tested the expression level of MEG3 in mice heart which undergone TAC or sham operation. The specific experimental methods in this step was in accordance with the previous study^21^. To demonstrate the correlations among MEG3, miR-361-5p and HDAC9, the expression levels of these three genes were separately tested in Sham and TAC group. MEG3 and HDAC9 were highly expressed in TAC group, while miR-361-5p was downregulated in TAC group (Fig. 5A). Next, the expression correlations among them were analyzed with Spearman’s correlation analysis. The negative relevance between MEG3 and miR-361-5p as well as between miR-361-5p and HDAC9 were analyzed (Fig. 5B). Additionally, the positive correlation between MEG3 and HDAC9 was analyzed (Fig. 5B).

### MEG3 improves the hypertrophy of cardiomyocyte by altering signals of miR-361-5p/HDAC9 axis

**Figure 6:**
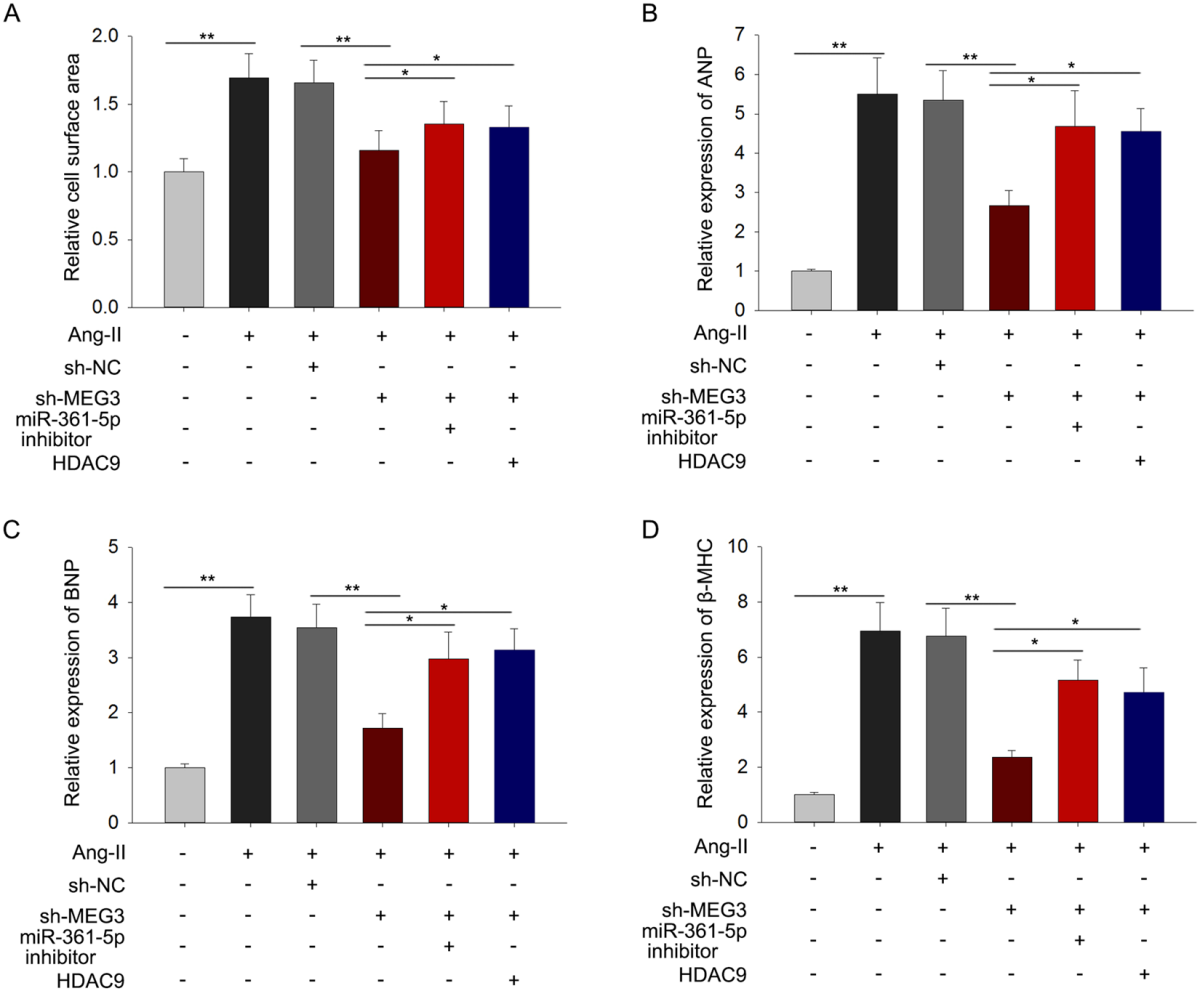
MEG3 improves the hypertrophy of cardiomyocyte by altering signals of miR-361-5p/HDAC9 axis. Recue assays were made after transfection for 48 hours. (**A**) The immunofluorescence staining was used to detect the cell size after co-transfected with sh-NC, sh-MEG3, miR-361-5p, and pcDNA-HDAC9 in both control group and Ang-II group (n = 3 per group; ^**^p < 0.01). Cell size was measured in 10 fields/well in both groups. (**B**–**D**) The expression levels of β-MHC, BNP and ANP were separately tested in control group and Ang-II group after the same transfections (n = 3 per group; ^**^P < 0.01).

Based on the previous results, we could found the relevance among MEG3, miR-361-5p and HDAC9. In order to confirm the exact functions of this pathway in the progression of cardiac hypertrophy, rescue assays were designed and conducted in hypertrophic cardiomyocytes induced by Ang-II at 48 hours’ transfection. To demonstrate that sh-MEG3 is not cytotoxic, the transfection efficiencies of mock, sh-NC and sh-MEG3 were detected by qRT-PCR (Supplementary Fig 1). We found sh-MEG3 largely shrank the size of CH cells in comparison with sh-NC, while this tendency could be reversed by miR-361-5p inhibitors and pcDNA-HDAC9 (Fig. 6A). Next, the expression of β-MHC, BNP and ANP was also observed in response to Ang-II, the results were in accord with the former study (Fig. 6B–D). The decreased expression of these three elements was observed when MEG3 expression was interfered, while this tendency was changed over in both groups when CH cell was transfected into miR-361-5p inhibitors and pcDNA-HDAC9. According to all these results, we concluded that MEG3 improved the hypertrophy of cardiomyocyte by altering signals of miR-361-5p/HDAC9 axis.

### The schematic illustration of the function of STAT3-MEG3-miR-361-5p-HDAC9 axis in Ang-II-induced cardiac hypertrophy

**Figure 7:**
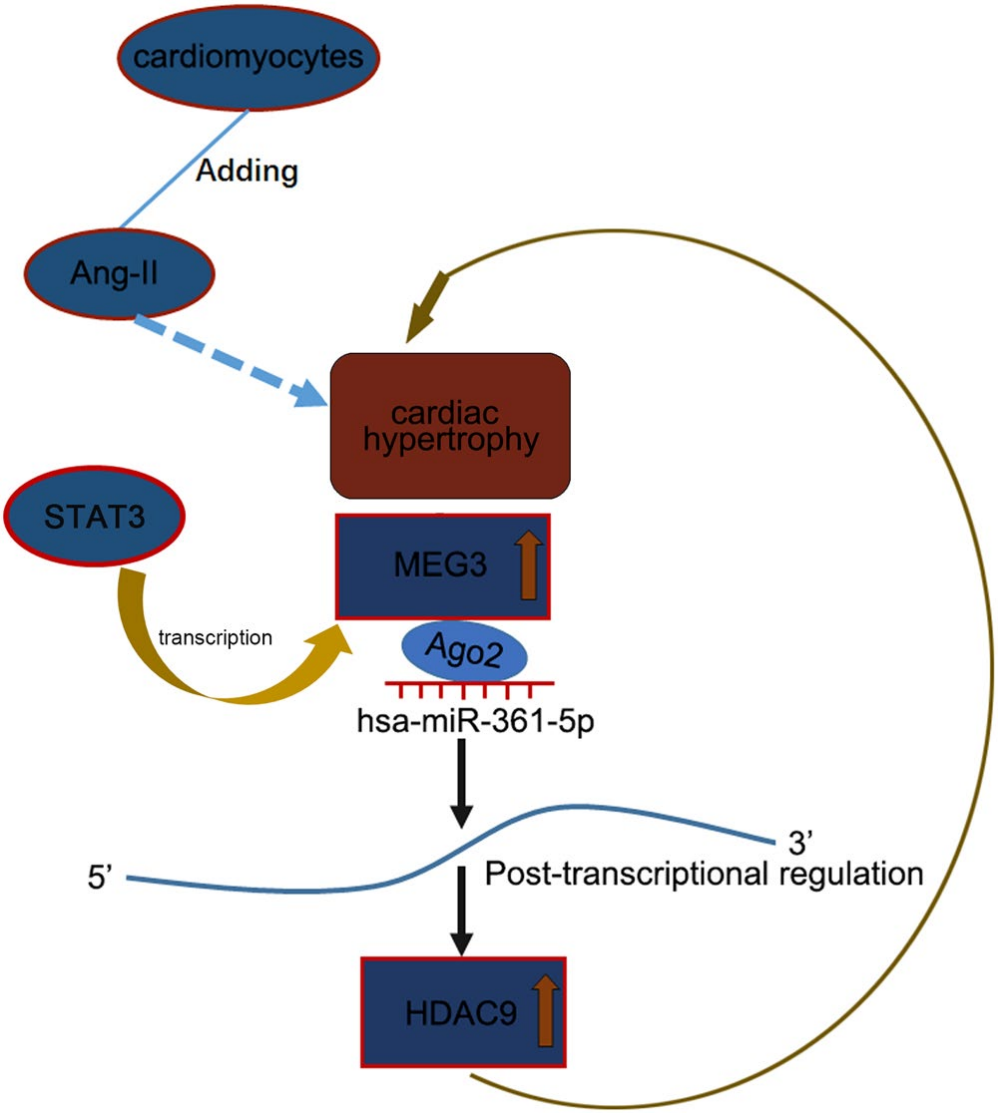
The schematic illustration of the function of STAT3-MEG3-miR-361-5p-HDAC9 axis in Ang-II-induced cardiac hypertrophy.

LncRNA MEG3 was highly expressed in Ang-II-induced CH cells. MEG3 was activated by STAT4 and promoted the hypertrophy in Ang-II-induced cardiomyocytes through miR-361-5p/HDAC9 axis (Fig. 7).

## Discussion

As a set of noncoding RNAs, lncRNAs were longer than 200nt. They have become the focus of researchers in the past decades. They are the vital molecule in researches because of their crucial role in complex biological process in human diseases^22^. It has been widely acknowledged that lncRNAs could act as regulators in human diseases. The role of them could be divided into two types: oncogene and tumor suppressor. It has been demonstrated that lncRNAs could regulate their downstream miRNAs to promote or suppress the occurrence and development of various diseases^23^. Growing number of lncRNAs have been verified to exert essential functions in heart failure^24–26^. While the study of the interaction between lncRNAs and cardiac hypertrophy still limited. Some lncRNAs have been certified to be a ceRNA in cardiac hypertrophy, such as lncRNA MIAT^6^ and lncRNA-GAS5^27^. Based on previous studies, we supposed MEG3 might be a ceRNA in CH.

MiRNAs are a group of RNA molecules which were coded by endogenous genes. They regulate genes at the post-transcriptional level. MiRNAs have been studied in human diseases, such as human cancers^28,29^ and cardiac hypertrophy^14^. It has been certified that miRNAs could be shared by lncRNAs and mRNAs to regulate biological behaviors of human diseases. As a member of miRNAs, miR-361-5p has been reported to be a suppressor in progression of human diseases by targeting mRNAs^30–33^. In this study, we found that miR-361-5p is a target gene of MEG3 through using bioinformatics analysis, pull-down assay and dual luciferase reporter assay. The reverse correlation between MEG3 and miR-361-5p was analyzed and demonstrated in CH. Therefore, we initially judged that MEG3 could competitively bind with miR-361-5p in CH. It is widely reported that mRNAs can act as downstream genes of miRNAs in ceRNA pathway. In a ceRNA pathway, lncRNAs regulate biological behaviors by sponging with miRNAs to regulate mRNA. MRNAs have been reported to be crucial factors to regulate the biological processes of human diseases^34,35^. HDAC9 was found to be a motivator in the progression of human diseases^36,37^. It also could be targeted by miRNA to alter progression of human diseases^38,39^. In this study, we found that HDAC9 was highly expressed in TAC and Ang-II group. The positive correlation between MEG3 and HDAC9 was demonstrated to support our hypothesis. The binding sites among MEG3, miR-361-5p and HDAC9 were obtained by using bioinformatics analysis. The exact combination was confirmed by pull down assay and luciferase activity assay. Finally, rescue assays were conducted to certify the function of MEG3-miR-361-5p-HDAC9 axis in CH progression. All findings in this study verified that lncRNA MEG3 was activated by STAT3 and positively regulated CH via miR-361-5p/HDAC9 axis (Fig. 7). These findings may help to develop novel therapeutic target for CH.

## Electronic supplementary material

Supplememtary Figure 1
