# Supplementary material for: STAT3-induced upregulation of lncRNA MEG3 regulates the growth of cardiac hypertrophy through miR-361-5p/HDAC9 axis
Source: Sci Rep. 2019 Jan 24;9:460. doi: 10.1038/s41598-018-36369-1 (PMC6346020; doi:10.1038/s41598-018-36369-1)
Supplement: Supplementary file 1 — Supplememtary Figure 1 [file 41598_2018_36369_MOESM1_ESM.pdf]

STAT3-induced upregulation of lncRNA MEG3 regulates the growth of cardiac hypertrophy through miR-361-5p/HDAC9 axis

Jingchang Zhang[1], Xuecheng Huang[1], Xiaoyan Guo[1], Yang Liu[1], Jiming Zhong[1], Jielin Yuan[1], Yi Liang[1, *]

[1]Department of Cardiology, The Third Affiliated Hospital of Guangxi Medical University, Nanning, Guangxi, 530031.

[*]**Address correspondence to:** Yi Liang, E-mail: Liangyii23@163.com

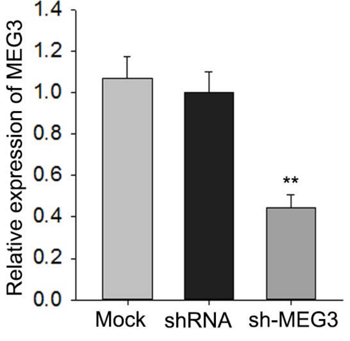
1.4
1.2
1.0
0.8
0.6
0.4
0.2
0.0
Relative expression of MEG3
**
Mock
shRNA
sh-MEG3
